# The efficacy of nursing protocol based on preoperative visualization rehearsal in the operating room, combined with intraoperative dynamic risk management and control in patients undergoing ambulatory hysteroscopy

**DOI:** 10.12669/pjms.42.5.14792

**Published:** 2026-05

**Authors:** Guoqin Chen, Xiaoxue Wang, Xueting Kan

**Affiliations:** 1 Guoqin Chen, Department of Operating Room,The first Affiliated Hospital of Zhejiang University, Traditional Chinese Medicine, Zhejiang Province, Chinese Medicine Hospital, Hangzhou, Zhejiang Province 310006, P.R. China; 2 Xiaoxue Wang, Department of Operating Room,The first Affiliated Hospital of Zhejiang University, Traditional Chinese Medicine, Zhejiang Province, Chinese Medicine Hospital, Hangzhou, Zhejiang Province 310006, P.R. China; 3 Xueting Kan, Department of Operating Room, The first Affiliated Hospital of Zhejiang University, Traditional Chinese Medicine, Zhejiang Province, Chinese Medicine Hospital, Hangzhou, Zhejiang Province 310006, P.R. China

**Keywords:** Ambulatory hysteroscopy, Adverse events, Intraoperative dynamic risk management and control, Nursing safety, Preoperative visualization rehearsal

## Abstract

**Objective:**

To explore the effect of preoperative visual rehearsal and intraoperative dynamic risk management in daytime hysteroscopy surgery.

**Methodology:**

In this retrospective study, the clinical data of 145 patients who underwent ambulatory hysteroscopy at the First Affiliated Hospital of Zhejiang University of Traditional Chinese Medicine from May, 2023 to May, 2025 were included. The patients were divided into two groups based on different nursing protocols: study group (74 patients who received the nursing protocol based on preoperative visualization rehearsal and intraoperative dynamic risk management in the operating room) and control group (71 patients who received routine perioperative nursing). Data on perioperative adverse events and nursing safety indicators were collected.

**Result:**

The incidence of perioperative adverse events in the study group was lower than that in the control group, the compliance rate of nursing safety indicators was higher than that in the control group, the duration of rehabilitation related indicators was shorter than that in the control group, SAS and SDS were lower than those in the control group, and nursing satisfaction was higher than that in the control group (P<0.05).

**Conclusion:**

Based on preoperative visualization rehearsal in the operating room and intraoperative dynamic risk management, adverse events during hysteroscopy surgery can be reduced and nursing safety can be improved.

## INTRODUCTION

Ambulatory hysteroscopy has become increasingly widely used for the diagnosis and treatment of benign gynecological diseases due to its minimally invasive nature and rapid recovery.^1^-^3^ However, as ambulatory hysteroscopy requires limited preoperative evaluation time and the surgical operation involves delicate intrauterine manipulations, the incidence of perioperative adverse events still poses a certain risk, which not only affects the patient’s treatment experience but also poses severe challenges to nursing safety management.^4^-^6^ Therefore, optimizing the perioperative nursing model and establishing a precise, comprehensive risk-prevention and control system are crucial for improving the quality of nursing care during ambulatory hysteroscopy.

The traditional nursing model of preoperative preparation and intraoperative management primarily relies on empirical evaluation and routine processes, which often lead to insufficient preoperative risk prediction, delayed intraoperative emergency response, delayed rehabilitation, and reduced safety for ambulatory surgery patients.^7^-^9^ In recent years, integrating visualization technology and dynamic risk management concepts into clinical nursing has generated new ideas for optimizing perioperative nursing.^10^,^11^ Preoperative visualization rehearsal, which combines 3D images generated by pelvic ultrasound and hysteroscopy with process simulation and related methods, helps the nursing team accurately formulate personalized nursing plans. At the same time, intraoperative dynamic risk management and control, relying on real-time monitoring, instant early warning, and other means, realizes dynamic tracking and timely intervention of risk factors during the surgical process, forming a closed-loop nursing management model of preoperative prediction - intraoperative intervention - whole-process management are important.^10^,^11^

At present, studies of an integrated nursing protocol of preoperative visualization rehearsal combined with intraoperative dynamic risk management and control for patients undergoing ambulatory hysteroscopy are scarce. This study aimed to systematically explore the impact of the preoperative visualization rehearsal combined with intraoperative dynamic risk management and control on the incidence of perioperative adverse events and nursing safety in patients undergoing ambulatory hysterectomy.

## METHODOLOGY

In this retrospective study, the clinical data of 145 patients who underwent ambulatory hysteroscopy at the First Affiliated Hospital of Zhejiang University of Traditional Chinese Medicine from May, 2023 to May, 2025 were retrospectively included and divided into two groups based on different nursing protocols: study group (74 patients who received the nursing protocol based on preoperative visualization rehearsal in the operating room + intraoperative dynamic risk management and control) and control group (71 patients who received routine perioperative nursing).

### Ethical approval:

The ethics committee of our hospital approved the study with the number: 2023L036; Date: April 23, 2023.

### Inclusion criteria:

- Aged 18-70 years old.
- Meeting the indications for ambulatory hysteroscopy and scheduled for elective surgery.
- American Society of Anesthesiologists (ASA) classification Grade I-II.
- Clear diagnosis by pelvic ultrasound, routine vaginal secretion examination and other preoperative examinations.
- Complete clinical data.

### Exclusion criteria:

- Uncontrolled hypertension, diabetes mellitus, severe dysfunction of important organs such as heart, liver and kidney.
- Abnormal coagulation function.
- Acute or subacute genital tract inflammation, active massive uterine bleeding.
- History of uterine perforation within the past three months.
- Narrow and rigid cervical canal difficult to dilate, abnormally small uterine cavity.
- Complicated with invasive cervical cancer.
- Cognitive impairment or history of mental illness.

### Nursing approaches

### Routine perioperative nursing:

### Preoperative nursing:

After admission, basic vital sign monitoring, medical history collection, and verification of preoperative examinations (pelvic ultrasound, blood routine, and coagulation function) were completed; verbal health education on surgical procedures, precautions, and postoperative rehabilitation knowledge was provided to patients; routine skin preparation and vaginal irrigation were performed; patients were instructed to fast and abstain from drinking before surgery, and psychological comfort was given to relieve tension.

### Intraoperative nursing:

After entering the operating room, patients were assisted to assume a comfortable surgical position, intravenous access was established, and anesthesia induction was coordinated with anesthesiologists; strict aseptic operation was implemented during surgery, vital signs were closely monitored, surgical instruments were delivered on time, and surgeons were assisted in completing the operation; in case of sudden abnormalities, handling was coordinated according to routine emergency plans.

### Postoperative nursing:

After surgery, patients were assisted to wake up and transferred to the recovery room for 0.5 hours of observation, with vital signs, vaginal bleeding, and abdominal pain monitored; patients were instructed to take liquid diet six hours after surgery, gradually transitioning to a normal diet; postoperative precautions (e.g., avoiding strenuous exercise, refraining from sexual activity for one month, etc.) were explained; patients’ recovery status was evaluated, discharge procedures were handled when discharge criteria were met, and follow-up time after discharge and handling methods for abnormal situations were informed.

### A nursing protocol based on preoperative visualization rehearsal in the operating room + intraoperative dynamic risk management and control:

### Preoperative visualization rehearsal nursing - Multidimensional visualization evaluation:

The preoperative visualization rehearsal was conducted by the same group of well-trained nursing staff as follows. At first, combined with the patient’s pelvic ultrasound and hysteroscopy 2D images, medical visualization software (Model: Stryker HD1288) was used to construct a 2D anatomical model of the uterine cavity, presenting key information such as uterine position, lesion size and location, and degree of intrauterine adhesions. It took 15-30 minutes for each patient. Then, preoperative discussions were conducted jointly with physicians and anesthesiologists to clarify surgical operation paths, potential risk points (e.g., uterine perforation, bleeding, water intoxication) and response key points based on the visualization model.

### Personalized rehearsal and health education:

According to the patient’s individual conditions, a visualized animation of the surgical process (including position placement, surgical steps, and instrument use) was produced. One day before the operation, the procedure was demonstrated to the patient and their family via a tablet, with simultaneous explanations of intraoperative cooperation points and risk prevention and control measures, and questions were answered to strengthen cognition; the nursing team was organized to conduct simulated rehearsals, and a personalized nursing cooperation plan was formulated based on the visualization model, clarifying the responsibilities of each post (e.g., key operation points for scrub nurses and circulating nurses).

### Optimization of preoperative preparation:

Based on the results of visualization evaluation, targeted emergency items (e.g., hemostatic instruments, special consumables for preventing uterine perforation, etc.) were prepared in advance; for patients at risk of cervical canal stenosis, cervical dilation preparation and nursing plans were made in advance; patient information and surgical plans were re-verified to ensure consistency between preoperative preparation and the visualization rehearsal plan.

### Intraoperative dynamic risk management and control nursing:

### Real-time monitoring and early warning:

A multi-functional monitoring system was adopted during surgery to continuously monitor the patient’s vital signs, uterine cavity pressure, inflow and outflow volume of irrigation fluid, and electrolyte levels. Risk early warning thresholds were set (e.g., automatic alarm when uterine cavity pressure >150mmHg or irrigation fluid difference >1000ml); the circulating nurse recorded monitoring data every five minutes, compared the surgical progress in real time with the visualization model, and predicted risk trends.

### Precise intraoperative cooperation:

According to the operation path and lesion characteristics from preoperative visualization rehearsal, the scrub nurse prepared corresponding instruments in advance and placed them in a standardized manner, accurately cooperating with the surgeon to complete lesion resection, adhesion separation and other operations, to shorten the surgical time; if early warning signals or emergencies occurred (e.g., bleeding >50ml, abnormal heart rate), the dynamic emergency plan was activated immediately, and the nurse quickly cooperated with the physician to implement hemostasis, fluid replacement and other treatments according to the pre-rehearsed division of labor.

### Real-time risk communication:

An intraoperative multidisciplinary communication mechanism was established. The nursing team synchronized risk information with the surgeon and anesthesiologist every 15 minutes and adjusted the nursing strategy based on monitoring data and surgical progress; potential risks (e.g., patient’s blood pressure fluctuations) were recorded and fed back promptly to ensure timely and accurate risk management.

### Postoperative continuous nursing:

In addition to routine postoperative nursing, targeted observation was strengthened based on preoperative rehearsal and intraoperative risk points: Focus on monitoring vaginal bleeding and abdominal pain in high-risk patients (e.g., those with significant intraoperative bleeding), and extend the vital sign monitoring time; re-strengthen key postoperative rehabilitation points through visualized health education materials, and provide personalized discharge guidance for patients and their families; conduct telephone follow-ups three days, one week, and one month after discharge to track recovery status, answer questions promptly, and reduce the risk of recurrence of postoperative adverse events.

### Observation Indicators:

The following indicators were collected from all patients The primary outcome was the incidence of perioperative adverse events. The secondary outcomes were compliance rate of core nursing safety, perioperative rehabilitation-related time, negative emotions before and after intervention, and nursing satisfaction.

### Incidence of perioperative adverse events:

The occurrence of adverse events during the perioperative period and within 14 days after the operation was counted in both groups, including uterine perforation, intraoperative massive bleeding (blood loss >100ml), uterine cavity infection, irrigation fluid absorption syndrome, severe postoperative abdominal pain (visual analog scale (VAS) score ≥7), and prolonged vaginal bleeding (>7 days).

Compliance rate of core nursing safety indicators, including the completion rate of preoperative preparation (complete examination verification and adequate risk prediction), standardization rate of intraoperative operations (aseptic operation and timely emergency cooperation), and qualification rate of postoperative management (standard vital sign monitoring and early identification of complications).

### Perioperative rehabilitation-related indicators:

The surgical operation time, postoperative awakening time, observation time in the recovery room, time to first ambulation after surgery, anal exhaust time, and total length of hospital stay were recorded for both groups.

### Negative emotions before and after intervention:

The Self-Rating Anxiety Scale (SAS) and Self-Rating Depression Scale (SDS) were used to evaluate emotions at admission (before intervention) and one hour before surgery (after intervention). Both scales consist of 20 items, with a total score of 100 points; a higher score indicates more severe anxiety or depression.

### Nursing satisfaction:

A dedicated scale (Cronbach’s α coefficient = 0.89) was designed using a Likert 5-point scoring method, with 20 items across 4 dimensions: clarity of risk notification, nursing professionalism, adequacy of preoperative preparation, and comfort of intraoperative care, with a total score of 100 points. Scores ≥90 were defined as “very satisfied”, 75-89 as “satisfied”, 60-74 as “fair”, and <60 as “dissatisfied”; the total satisfaction rate was calculated as (number of very satisfied + satisfied cases) / total number of cases × 100%.

### Statistical analysis:

Data were analyzed using SPSS 25.0 software. Measurement data were expressed as (±s) and compared by t-test; enumeration data were expressed as frequency and composition ratio (%) and compared by χ² test. A P value <0.05 was considered statistically significant.

## RESULTS

There were no statistically significant differences in baseline data, including age, body mass index (BMI), educational level, surgical type, and ASA classification, between the two groups (P>0.05), indicating comparability Table-I.

**Table-I:** Comparison of Baseline Data Between the Two Groups.

Item	Study Group (n=74)	Control Group (n=71)	t/χ^2^	P
Age (±s, years)	51.26±7.35	50.83±6.52	0.372	0.710
Body Mass Index (±s, kg/m²)	24.12±1.63	23.75±1.97	1.234	0.219
***Educational Level [n (%)]***				
Primary School and Below	8(10.81)	6(8.45)	0.423	0.935
Junior High School	19(25.68)	18(25.35)		
Senior High School/Secondary Vocational School	22(29.73)	20(28.17)		
College and Above	25(33.78)	27(38.03)		
***Surgical Type [n (%)]***				
Endometrial Polyp Resection	33(44.59)	30(42.25)	0.842	0.940
Submucous Uterine Fibroid Resection	19(25.68)	18(25.35)		
Intrauterine Adhesion Separation	16(21.62)	14(19.72)		
Others	6(8.11)	9(12.68)		
***ASA Classification[n(%)]***				
Grade-I	45(60.81)	41(57.75)	0.141	0.707
Grade-II	29(39.19)	30(42.25)		

The incidence of perioperative adverse events in the study group (5.41%) was lower than that in the control group (18.31%) (P<0.05). In Table-II, the completion rate of preoperative preparation (97.30%), standardization rate of intraoperative operations (95.95%), and qualification rate of postoperative management (94.59%) in the study group were higher than those in the control group (88.73%, 85.92%, 84.51%) (P<0.05 Table-III.

**Table-II:** Comparison of Perioperative Adverse Event Incidence Between the Two Groups [n (%)].

Group	No. of Cases	Uterine Perforation	Intraoperative Massive Bleeding	Uterine Cavity Infection	Irrigation Fluid Absorption Syndrome	Severe Postoperative Abdominal Pain	Prolonged Vaginal Bleeding	Total Incidence
Study Group	74	0(0.00)	1(1.35)	1(1.35)	0(0.00)	1(1.35)	1(1.35)	4(5.41)
Control Group	71	2(2.82)	3(4.23)	2(2.82)	1(1.41)	3(4.23)	2(2.82)	13(18.31)
*χ^2^*								6.009
*P*								0.014

**Table-III:** Comparison of Compliance Rates of Core Nursing Safety Indicators Between the Two Groups[n(%)].

Group	No. of Cases	Completion Rate of Preoperative Preparation	Standardization Rate of Intraoperative Operations	Qualification Rate of Postoperative Management
Study Group	74	72 (97.30)	71 (95.95)	70 (94.59)
Control Group	71	63 (88.73)	61 (85.92)	60 (84.51)
*χ^2^*		4.140	4.467	3.976
*P*		0.042	0.035	0.046

The surgical operation time, postoperative awakening time, post-anesthesia care unit (PACU) observation time, time to first ambulation after surgery, anal exhaust time, and total hospital stay in the study group were shorter than those in the control group (P<0.05; Table-IV There were no significant differences in SAS and SDS scores between the two groups before intervention (P>0.05). After intervention, the SAS and SDS scores of both groups decreased compared with pre-intervention levels, and the scores in the study group were markedly lower than those in the control group (P<0.05) Table-V. Similarly, the nursing satisfaction rate in the study group (94.59%) was higher than that in the control group (83.10%) (P<0.05; Table-VI.

**Table-IV:** Comparison of Perioperative Rehabilitation-Related Indicators Between the Two Groups (*x̄*+*s*).

Group	No. of Cases	Surgical Operation Time (min)	Postoperative Awakening Time (min)	PACU Observation Time (h)	Time to First Ambulation After Surgery (h)	Anal Exhaust Time (h)	Total Hospital Stay (h)
Study Group	74	35.36±4.28	8.24±2.15	2.35±0.42	4.26±1.05	6.35±1.24	18.52±3.16
Control Group	71	46.04±6.09	11.36±3.87	3.12±0.58	6.15±1.32	8.42±1.56	22.48±5.85
*t*		12.258	6.033	9.184	9.562	8.864	5.100
*P*		<0.001	<0.001	<0.001	<0.001	<0.001	<0.001

**Table-V:** Comparison of Negative Emotions Between the Two Groups (*x̄*+*s*, scores).

Time	Group	Number of Cases	SAS	SDS
Pre-intervention	Study Group	74	58.32±6.15	56.45±5.87
	Control Group	71	57.86±5.98	55.92±6.04
	*t*		0.456	0.536
	*P*		0.649	0.593
Post-intervention	Study Group	74	45.28±4.86	47.25±4.52
	Control Group	71	50.15±5.62	51.71±5.36
	*t*		5.588	5.425
	*P*		0.000	0.000

**Table-VI:** Comparison of Nursing Satisfaction between the Two Groups[n(%)].

Group	No. of Cases	Very Satisfied	Satisfied	Fair	Dissatisfied	Total Satisfaction Rate
Study Group	74	42 (56.76)	28 (37.84)	3 (4.05)	1 (1.35)	70 (94.59)
Control Group	71	30 (42.25)	29 (40.85)	7 (9.86)	5 (7.04)	59 (83.10)
*χ^2^*						4.878
*P*						0.027

## DISCUSSION

Currently, there is a scarcity of research on comprehensive nursing protocol of preoperative visualization rehearsal combined with intraoperative dynamic risk management and control for patients undergoing ambulatory hysteroscopy. Therefore, this study was conducted with a restrict eligibility criteria and detailed nursing protocol. The study showed that a nursing protocol based on preoperative visualization rehearsal in the operating room, combined with intraoperative dynamic risk management and control in patients undergoing ambulatory hysteroscopy, is associated with a lower incidence of perioperative adverse events, improved compliance with core nursing safety indicators, and accelerated perioperative rehabilitation. The combined approach effectively alleviates preoperative negative emotions and enhances nursing satisfaction.

Due to a short surgical cycle, delicate intrauterine operations, and limited preoperative evaluation time, the prevention and control of perioperative adverse events and nursing safety management have become key clinical concerns in patients undergoing ambulatory hysterectomy.^12^ In such patients, conventional perioperative nursing is associated with insufficient risk prediction and delayed intraoperative intervention.^13^

In this study, the incidence of adverse events in the study group was significantly lower than in the control group (P<0.05), which is consistent with previous research showing that visualization technology and dynamic risk management can reduce surgical risks.^14^,^15^ It is possible that preoperative visualization rehearsal, through the construction of 3D anatomical models and surgical process simulation, enables the nursing team to accurately grasp the individual characteristics of patients such as the location of intrauterine lesions and uterine morphology, predict potential risks such as uterine perforation and irrigation fluid absorption syndrome in advance, and prepare targeted emergency items and formulate intervention plans to reduce risk factors from the source. Intraoperative dynamic risk management and control rely on real-time monitoring and early warning systems to track key indicators, such as uterine cavity pressure and irrigation fluid inflow and outflow, enabling early identification and rapid mitigation of risks and preventing the progression of minor risks into severe adverse events. In contrast, the conventional nursing (control group) lacks personalized risk prediction and intraoperative monitoring. It primarily focuses on basic vital signs, with insufficient sensitivity to risks associated with intraoperative procedures, resulting in a persistently high incidence of adverse events.

Another important advantage of the protocol in this study is the improvement in the compliance rate of core nursing safety indicators. The completion rate of preoperative preparation, standardization rate of intraoperative operations, and qualification rate of postoperative management in the study group were significantly higher than those in the control group (P<0.05), indicating that the protocol can promote the transformation of nursing management from experience-driven to precision-driven. Preoperative visualization rehearsal, through multidisciplinary discussions and nursing team simulated drills, clarified the operational key points and personnel cooperation processes for each post, reducing problems such as omissions in preoperative examination verification and insufficient instrument preparation. The real-time communication mechanism established by intraoperative dynamic risk management and control strengthened collaboration among the nursing team, surgeons, and anesthesiologists, making the implementation of aseptic operations and emergency response procedures more standardized. Postoperative personalized management based on preoperative risk factors and intraoperative conditions improved the relevance and effectiveness of vital sign monitoring and early identification of complications, forming a closed-loop safety management system spanning preoperative, intraoperative, and postoperative periods and comprehensively improving the quality of nursing safety.^16^,^17^

In addition, the rehabilitation indicators, such as surgical operation time and total hospital stay, in the study group were shorter than those in the control group (P<0.05), indicating that visualization rehearsal and dynamic management optimization are closely related to improved surgical efficiency. The preoperative visualization model provided surgeons with intuitive anatomical references and operation path planning, reducing intraoperative exploration time and operational errors, and indirectly shortening the surgical duration. Precise intraoperative nursing collaboration reduced the likelihood of surgical interruption, while dynamic risk management and control minimized rehabilitation delays resulting from adverse events. Targeted postoperative rehabilitation guidance and observation encouraged patients to get out of bed earlier and to recover gastrointestinal function, consistent with the Enhanced Recovery After Surgery (ERAS) concept for ambulatory surgery.^18^,^19^ In contrast, the control group had more ineffective time consumption in the surgical and rehabilitation processes due to inaccurate preoperative preparation and a lack of personalized guidance for intraoperative cooperation, which affected the rehabilitation progress.

Furthermore, the results of this study indicate that alleviating patients’ negative emotions and improving nursing satisfaction reflect the humanistic care value of this nursing protocol. After intervention, the SAS and SDS scores of the study group were significantly lower than those of the control group, while the nursing satisfaction rate (94.59%) was higher (83.10%) (P<0.05). This benefit stems from the unique advantages of preoperative visualized health education: explaining the surgical process and risk prevention and control measures to patients through visualized methods effectively reduces anxiety and depression caused by information asymmetry, and enhances their trust in treatment and compliance. Effective intraoperative risk management, standardized nursing staff operations, and timely care improve patients’ medical experience. Personalized postoperative rehabilitation guidance and follow-up services further strengthen patients’ sense of gain and satisfaction. In contrast, the verbal health education adopted by the control group is relatively abstract, making it difficult to alleviate patients’ preoperative concerns effectively. Additionally, the standardization of the nursing process lacks personalized care, resulting in unsatisfactory outcomes in emotional improvement and satisfaction.

This study organically integrates preoperative visualization rehearsal with intraoperative dynamic risk management and control, thereby constructing a full-process nursing protocol tailored to the characteristics of ambulatory hysteroscopy. From a clinical perspective, it overcomes the limitation of disjointed preoperative and intraoperative management in the traditional nursing model, enabling precise, whole-process risk prevention and control.

### Limitations:

It is a single-center retrospective study with a limited sample size, and no stratified analysis of the intervention effect by surgical type was conducted, which may limit the generalizability of the results. In addition, multivariate analysis was not performed, so residual confounding may not be completely ruled out.

## CONCLUSION

The nursing protocol based on preoperative visualization rehearsal in the operating room + intraoperative dynamic risk management and control can achieve multiple goals for patients undergoing ambulatory hysteroscopy, including reduced perioperative adverse events, improved nursing safety, accelerated rehabilitation, alleviated negative emotions, and enhanced satisfaction. The combined protocol enables precise risk prevention and control, standardized nursing operations, and personalized, humanistic care. It meets the management needs of modern ambulatory surgery and may be promoted for routine clinical use.

### Recommendations:

In the future, multi-center prospective studies can be carried out to expand the sample size and refine the study grouping, further verify the clinical value of this protocol, and provide more sufficient evidence to support the optimization of the nursing model for ambulatory hysteroscopy.

### Authors’ contributions:

**GC:** Literature search, study design and manuscript writing.

**XW and XK:** Data collection, data analysis and interpretation. Critical Review.

**GC:** Manuscript revision and validation and is responsible for the integrity of the study.

All authors have read and approved the final manuscript.
